# Bridging the gap: A systematic review of intraoperative electrocochleography during cochlear implantation and preservation of residual hearing

**DOI:** 10.1371/journal.pone.0323493

**Published:** 2025-05-13

**Authors:** Jaimee Cooper, Jeenu Mittal, Max Zalta, Nicholas DiStefano, Delany L. Klassen, Keelin McKenna, Dimitri A. Godur, Andrea Monterrubio, Moeed Moosa, Rahul Mittal, Adrien A. Eshraghi

**Affiliations:** 1 Department of Otolaryngology, Hearing Research and Cochlear Implant Laboratory, University of Miami Miller School of Medicine, Miami, Florida, United States of America; 2 School of Medicine, New York Medical College, Valhalla, New York, United States of America; 3 Department of Neurological Surgery, University of Miami Miller School of Medicine, Miami, Florida, United States of America; 4 Department of Biomedical Engineering, University of Miami, Coral Gables, Florida, United States of America; 5 Department of Pediatrics, University of Miami Miller School of Medicine, Miami, Florida, United States of America; Universidad de Chile, CHILE

## Abstract

Cochlear implantation is a surgical intervention to provide auditory rehabilitation to individuals with severe to profound hearing loss. Intraoperative electrocochleography (ECochG) has emerged as a promising tool for monitoring cochlear health during cochlear implant (CI) surgery. This systematic review aims to synthesize current evidence regarding the effectiveness of intraoperative ECochG in predicting postoperative residual hearing levels in CI recipients. A comprehensive literature search was conducted across major databases including PubMed, Embase, Web of Science, and SCOPUS. The protocol for this systematic review was registered in the PROSPERO database (registration number: CRD42023476617). The key outcomes assessed were the correlation between intraoperative ECochG patterns and postoperative residual hearing levels, as well as the influence of surgical techniques and electrode design on ECochG responses and hearing preservation. The Risk of Bias analysis was conducted using the Joanna Briggs Institute Critical Appraisal Tool. The review included a total of eighteen studies that met the inclusion and exclusion criteria. A significant correlation was reported between specific intraoperative ECochG response patterns and the preservation of residual hearing post-surgery. Studies highlighted that robust ECochG responses typically indicated a higher likelihood of postoperative hearing preservation. The review also identified factors influencing ECochG responses, including electrode design and insertion techniques. Several studies reported improved preservation of residual hearing with modifications in surgical approaches guided by ECochG feedback. Intraoperative ECochG monitoring emerges as a crucial tool in predicting and potentially enhancing postoperative residual hearing outcomes in implanted individuals. The review underscores the value of ECochG in guiding surgical technique adjustments, thereby maximizing hearing preservation. However, the heterogeneity in study designs and ECochG protocols suggests a need for standardization in this field. Future research should focus on large-scale, multicenter trials to establish definitive guidelines for integrating ECochG in CI surgeries, with an emphasis on long-term hearing outcomes.

## Introduction

**Fig 1:**
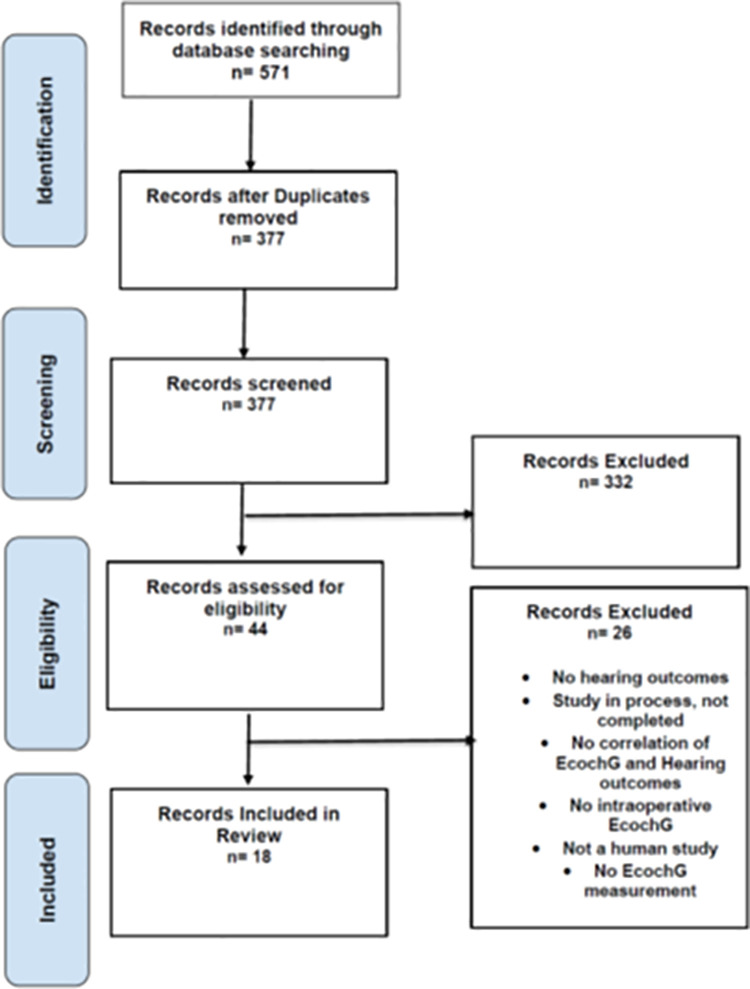
PRISMA flow diagram. This figure illustrates the PRISMA (Preferred Reporting Items for Systematic Reviews and Meta-Analyses) flow diagram showing the number of records identified, screened, included, and excluded throughout the systematic review process.

Cochlear implants (CIs) represent the gold standard in treatment for individuals with severe to profound hearing loss [1–9]. CIs capture environmental sounds through an external microphone, which are processed and converted into electrical signals by a speech processor [10,11]. These signals are then transmitted to an implanted electrode array in the cochlea. The electrode stimulates the auditory nerve, bypassing damaged hair cells located on the organ of Corti (OC) in the inner ear, sending sound information directly to the brain, enabling sound perception for the implanted individual [10,11]. As implant technologies continue to advance, their versatility and efficacy have improved, though they are not without limitations. One of the major challenges is the preservation of residual (natural) hearing following cochlear implantation [12–18]. Residual hearing not only offers a sense of safety with awareness of loud environmental sounds when the processor is offline but also opens up opportunities for integrating novel electroacoustic systems (EAS), which can enhance hearing results [19–21]. In addition, it facilitates better speech understanding, especially in noisy environments [22–24]. Consequently, preserving residual hearing during the CI surgery is a high priority research area. Intraoperative monitoring stands out as a promising approach to provide immediate feedback on the electrode insertion and potential residual hearing outcomes. In this context, electrophysiological methods, especially electrocochleography (ECochG), are gaining prominence as potential monitoring systems during CI surgeries [25–30]. This systematic review focuses on exploring the application of intraoperative ECochG monitoring in CI surgeries and its relationship with the preservation of residual hearing postoperatively. The central hypothesis is that larger amplitude and consistent intraoperative ECochG readings contribute to minimizing trauma during the CI insertion process, which in turn, is expected to result in better preservation of hearing. This review critically assesses various ECochG measurement and analysis techniques, examining their dependability and impact on the maintenance of hearing levels after the implantation (Fig 1).

**Fig 2:**
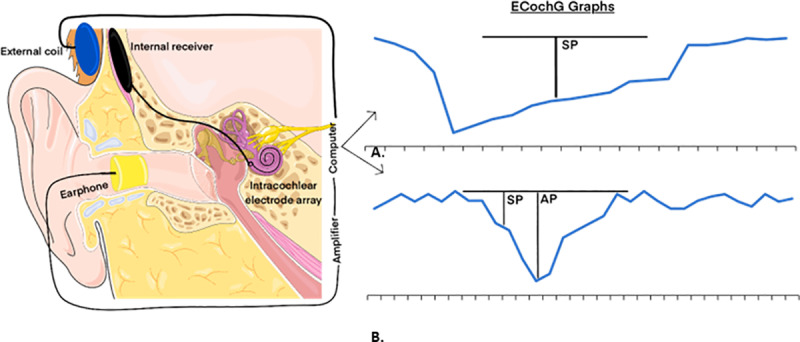
A schematic representation of intraoperative electrocochleography (EcochG). Panel A displays a graphical representation of the outcomes from a click burst EcochG measurement, whereas panel B shows a graphical representation from a tone burst EcochG recording. The charts include two key components: the summating potential (SP) and the action potential (AP). The figure was generated using images from Servier Medical Art, provided by Servier, licensed under a Creative Commons Attribution 4.0 unported license.

**Fig 3:**
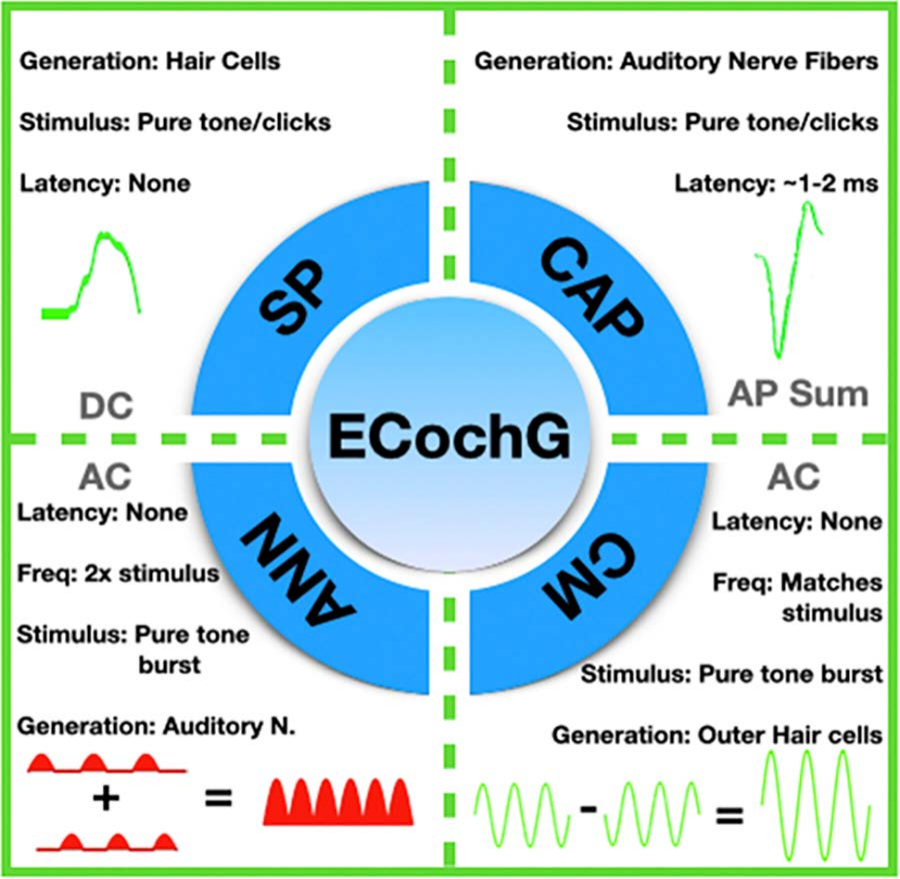
The four main components of intraoperative electrocochleography (EcochG). Cochlear microphonics (CM) and auditory nerve neurophonics (ANN) are alternating currents elicited from a tone burst stimuli of alternating polarity. CM represents a mechanical signal from the outer hair cells. ANN represents the phase-locked signal from the auditory nerve. The Summating Potential (SP) and the Compound Action Potential (CAP) are direct currents. SP represents responses from all hair cells, while the CAP represents responses from the auditory nerve. Taken from Barnes et al. [51] under a Creative Commons license.

ECochG captures the electrical potential generated within the inner ear and auditory nerve [31] (Fig 2). This is achieved by playing a short auditory stimulus and recording the physiological response through various types of electrodes, including skin electrodes, extra tympanic electrodes, intratympanic electrodes, or intracochlear electrodes connected to a CI [32]. Historically, ECochG has played a role in diagnosing Meniere’s disease and other inner-ear conditions [33]. However, its application has been expanded to include real-time usage during CI surgeries. The responses recorded in ECochG are classified into four distinct categories: cochlear microphonics (CM), auditory nerve neurophonics (ANN), summating potential (SP), and compound action potential (CAP) (Fig 3) [34]. Cochlear microphonics (CM) represent the electrical responses generated predominantly by the outer hair cells of the cochlea in reaction to acoustic stimuli. These responses are typically recorded by delivering auditory stimuli, such as clicks or tone bursts, and capturing the resulting potentials through electrodes placed at strategic locations. For intraoperative ECochG, electrodes can be positioned on the skin, within the ear canal, or intracochlearly via the cochlear implant array itself. The CM response closely follows the waveform of the acoustic stimulus, reflecting the biomechanical motion of the basilar membrane and the functioning of outer hair cells in specific regions of the cochlea. Modern acquisition techniques involve advanced signal processing to isolate the CM from other components of the ECochG signal, such as auditory nerve neurophonics (ANN) or compound action potentials (CAP), which overlap temporally. Interpreting CM involves assessing parameters like amplitude and phase consistency across the frequency spectrum, as these characteristics provide insights into cochlear integrity and potential trauma during electrode insertion. High-amplitude, stable CM recordings are generally indicative of minimal mechanical disruption, while abrupt decreases or irregularities may signal trauma or damage, underscoring its utility in guiding surgical adjustments in real time. By providing a dynamic representation of cochlear health during implantation, CM measurements enhance the surgeon’s ability to optimize electrode placement and preserve residual hearing. Current evidence indicates that CM is the most reliable indicator of trauma during the insertion of a CI [32]. The ECochG recordings along an electrode’s length are indicative of the specific regions, stimulated by the sound, which correspond to different sound frequencies. Consequently, assembling multiple recordings offers a comprehensive view of the condition across the entire stimulable area [35]. Modern techniques facilitate two forms of ECochG recordings: continuous and segmented. Continuous recordings provide ongoing, real-time feedback to surgeons during cochlear implant insertion, allowing them to monitor cochlear responses as the electrode array is advanced. In contrast, segmented recordings are obtained at predefined intervals rather than continuously during the insertion process, offering snapshots of cochlear responses at specific stages of the procedure. These recordings assist in identifying potential trauma and optimizing surgical techniques. Additionally, to assess hearing levels, audiological evaluations such as auditory brainstem response (ABR) measurements and pure-tone behavioral audiograms are performed at various intervals both preoperatively and postoperatively, complementing intraoperative monitoring data.

## Methods

### Search strategy

This systematic review was conducted in accordance with the Preferred Reporting Items for Systematic Reviews and Meta-Analyses (PRISMA). The protocol for this systematic review was designed *a priori* and was registered in the PROSPERO database (registration number: CRD42023476617). A literature search was performed in PubMed, Embase, Web of Science, and SCOPUS databases using the MeSH terms: “intraoperative electrocochleography” OR “intraoperative ECochG”, “electrocochleography AND cochlear implant surgery”, “ECochG feedback during CI Procedure” “cochlear implant”, “intraoperative electrocochleography and hearing outcomes during CI surgery”, “postoperative residual hearing” OR “postoperative hearing preservation”, and “ “ECochG Monitoring in CI Surgery”.

### Study selection

A team of reviewers (D.G., D.K., M.Z., J.M., K.M., M.M, A.M., and J.C.) independently reviewed all searched articles, abstracts, and full-text publications. Any disagreements on the exclusion or inclusion of results were resolved by another reviewer or senior author. Study inclusion criteria includes: cochlear implant recipient, electrocochleography, and preoperative and postoperative hearing assessment. Study exclusion criteria include: preoperative or postoperative hearing assessment, publication not in a peer-reviewed article, reviews, commentaries, conference proceedings, case reports/studies, non-human studies, ex-vivo or in-vitro studies, and studies not originally published in English.

### Handling of missing data

In this systematic review, we applied rigorous inclusion and exclusion criteria to ensure the selection of studies with complete and reliable data. During the screening process, studies were assessed for completeness, and those with insufficient or missing critical data relevant to our analysis were excluded. Specifically, we required all included studies to provide comprehensive information on study design, sample size, intervention details, and outcome measures. In cases where a study lacked minor data points but still met the overall inclusion criteria, we carefully assessed whether the missing information would impact on the validity of our findings. However, no included studies had missing data that compromised the integrity of our review. As a result, no additional imputation methods or sensitivity analyses were required. This approach ensured that our systematic review was based on high-quality, complete datasets, minimizing the risk of bias and enhancing the reliability of our conclusions.

### Quality assessment

The Risk of Bias (RoB) analysis was conducted using the Johanna Briggs Institute (JBI) Critical Appraisal Tool. The appropriate checklist was utilized based on the type of study. This assessment was completed by at least four reviewers (D.G., D.K., M.Z., J.M., and J.C) independently, with discrepancies resolved by discussion and consensus or discussion with the senior author.

### Data extraction

A team of reviewers (D.G., D.K., M.Z. and J.C) independently reviewed included articles. The information gathered includes: study type, population, comparison/study, ECochG system used, hearing thresholds, and outcomes/conclusions.

## Results

### Search results

Our initial search identified 571 articles relevant to our study. Through a deduplication process, we reduced this number to 377 unique articles, eliminating redundancies. We then rigorously screened titles and abstracts against our research criteria, narrowing the selection to 44 articles. The most critical phase involved a full-text review against specific inclusion and exclusion criteria, focusing on aspects such as study design and methodological quality. This comprehensive evaluation resulted in 18 articles that met all criteria and were included in our systematic review. The detailed search strategy using PRISMA flowchart is shown in Fig 1.

### Risk of bias analysis

**Fig 4:**
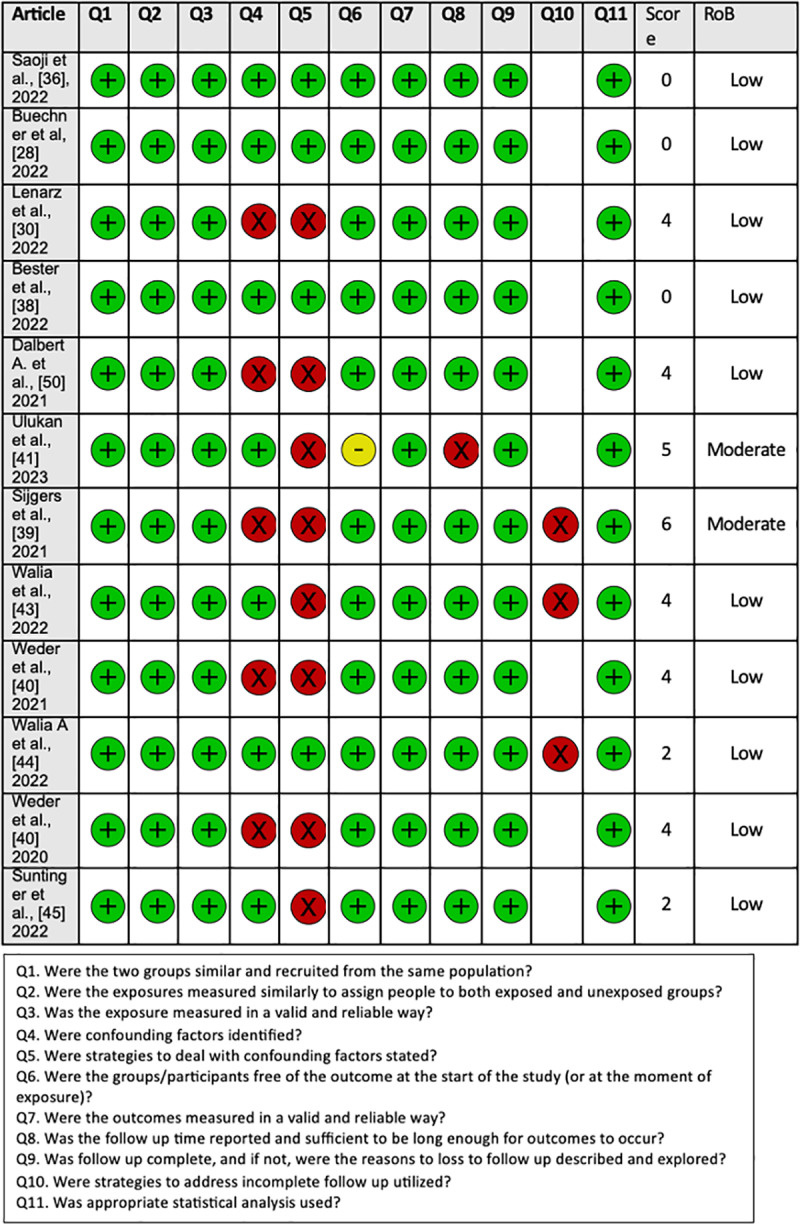
The Joanna Briggs Institute (JBI) risk of bias assessment for cohort studies.

**Fig 5:**
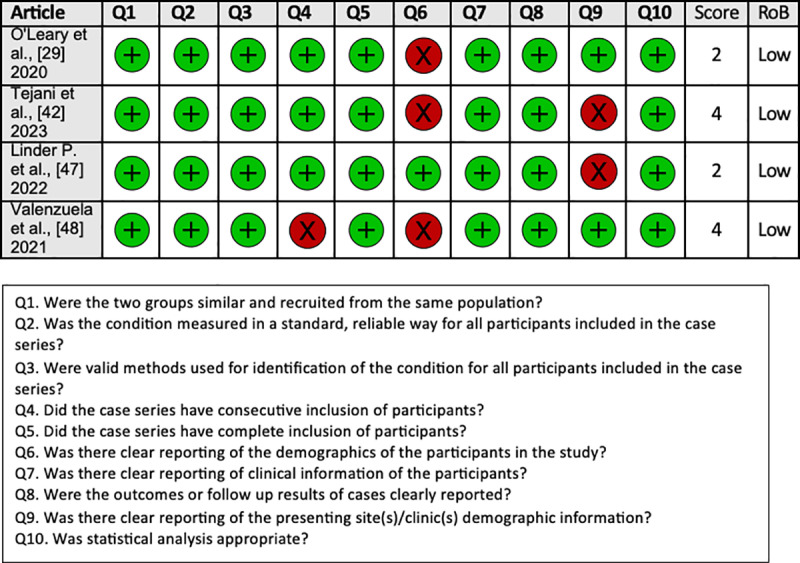
The Joanna Briggs Institute (JBI) risk of bias assessment for case series.

**Fig 6:**
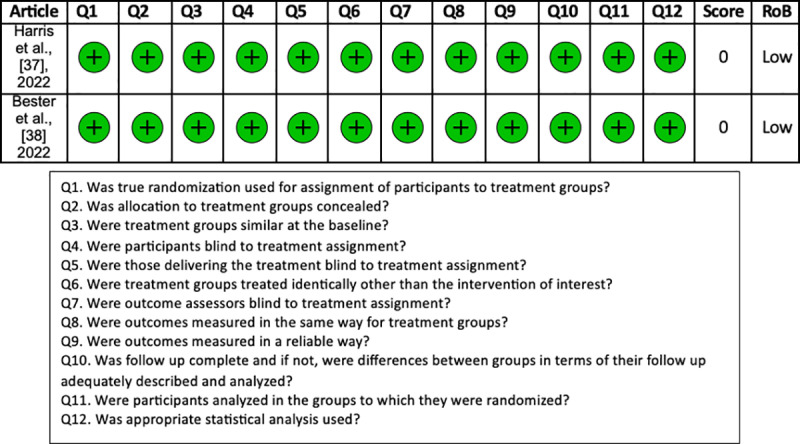
The Joanna Briggs Institute (JBI) risk of bias assessment for randomized control trials.

The RoB assessment conducted on the 18 studies included in our systematic review revealed a low risk of bias, indicating their overall good quality. The RoB assessment of included studies showed that almost all 18 studies had a low risk of bias and were of good quality. The detailed RoB quality assessment for cohort studies, case series, and RCTs assessed with the JBI critical appraisal tool are shown in Figs 4, 5, and 6, respectively.

### Study characteristics

There are 18 studies included with a total of 944 patients receiving CIs. Seventeen out of eighteen included studies focused on only adults, with the other including patients aged 5–20 (which the authors classified as children). Sixteen out of eighteen articles recorded electrocochleography intraoperatively, with one of these sixteen recording pre- and post-insertion, but not during insertion. Two studies only recorded postoperatively. A detailed summary of the included studies has been shown in Table 1.

**Table 1:** A summary of included studies.

Reference	Study Type	Population	Exposure	Outcomes
Saoji et al., [36] 2022	Prospective cohort	Sixteen participants with preoperative residual hearing and bone conduction thresholds at 200 or 500 Hz who got Cis.	CI	Nine patients had a small drop in ECochG amplitude (<30%) with 46dB preoperative and 39dB postoperatively at 500Hz on average. Those with a > 30% drop in ECochG amplitude had, on average, 32dB preop and 55dB postoperative at 500Hz.
Harris et al., [37] 2022	Prospective randomized clinical trial	Eighty-five adults with SNHL who meet criteria for CI and chose an Advanced Bionics CI.	CI	Average preoperative pure tone average was 54dB. PTA change was good (0-15dB) in 34.5%, fair (>15-29dB) in 23.5%, and poor (>30dB) in 43% of participants. Mean PTA change in both groups was 27dB, thus showing no difference in the hearing preservation with and without the use of ECochG. This study found a significant decrease in translocations
Buechner et al., [28] 2022	Prospective cohort	Sixty-four subjects over the age of 18 who received an Advanced Bionics implant and who had measurable residual hearing.	CI	Subjects with a consistent increase in ECochG potentials during insertion had the smallest loss of acoustic hearing postoperatively. Patients with fluctuating electrocochleography results had no clear correlation.
Lenarz et al., [30] 2022	Prospective cohort	Sixty-eight adult CI candidates who received either a Mid-Scala or SlimJ Advanced Bionics implant with preoperative hearing thresholds < 90dB at 500Hz.	CI	Changes in ECochG less than 1 dB had PTA changes from 0–30 db. Larger ECochG drops had larger changes in PTA. This correlation was significant in SlimJ electrodes.
Bester et al., [38] 2022	Single-blinded placebo-controlled trial	Sixty adults with Cochlear Ltd Thin straight electrode.	CI	Hearing preservation was significantly better in the intervention (pull back) than the control group. Speech in noise perception thresholds were significantly better in patients with no CM drop during insertion. Larger cochlear size was associated with better CM recovery.
Sijgers et al., [39] 2021	Prospective cohort	Six Adults undergoing CI implantation at the University of Zurich Hospital.	CI with ECochG	Decreased intracochlear ECochG recordings early in insertion are not always seen on extracochlear recordings, this may be due to the movement of the recording electrode in relation to the signal generators. Amplitude changes later in insertion are generally reflected in extracochlear ECochG and may represent trauma to the cochlea.
Weder et al., [40] 2020	Prospective cohort	Fifty-five patients with post-lingual hearing loss who received CI522 slim straight electrode and had preoperative hearing threshold of 100 dB or less at 500Hz.	CI	Loss of 61% of the DIF signal was the best cut-off criterion for association with 25% postoperative hearing loss (25% relative loss on the audiogram).
O’Leary et al., [29] 2020	Prospective cohort	One hundred and nine patients with <80-db HL at 500 Hz.	CI	Drop in ECochG amplitude during surgery was associated with greater loss of hearing (.25,.5, 1 kHz during the first 12 months after implantation). Sudden drop in signal amplitude was seen in > 30% of the real-time intraoperative recordings.
Bayri Ulukan et al., [41] 2023	Prospective cohort study	Sixty-two implanted ears in 46 recipients. Twenty-nine with Auditory neuropathy spectrum disorder and thirty-three with SNHL.	CI	There was a difference between the ANSD and SNHL patients in hearing threshold. CM thresholds obtained by the ECochG don’t necessarily reflect behavioral hearing thresholds.
Tejani et al., [42] 2023	Prospective cohort	Forty subjects with CI, split into two groups (stable acoustic hearing, loss of acoustic hearing).	CI	ECochG thresholds and amplitudes were stable in those with residual hearing, but thresholds worsened for those that started with baseline hearing loss.
Walia et al., [43] 2022	Prospective cohort study	Forty-eight adult CI candidates.	CI	ECochG CM drops using 250 Hz stimulus were more correlated with the audiogram shifts than the ECochG CM changes using 500 Hz stimulus.
Walia et al., [44] 2022	Prospective cohort study	Thirty-five adult patients that received CI in a single center.	CI	ECochG had a moderate to strong correlation with the low frequency pure tone average and mean pure tone average.
Suntinger et al., [45] 2022	Prospective cohort	Sixty-eight subjects.	CI	ECochG signals are a feasible tool for detecting cochlear trauma. Postoperative PTA was associated with phase changes: increase of > 90 degrees correlated with significantly larger change in PTA. Postoperative PTA was also associated with increased ECochG amplitude, > 3dB increase correlated with PTA change of 16 dB. Preoperative PTA was not correlated with phase or amplitude change
Bester et al., [46] 2022	Prospective cohort	Thirty-nine subjects with residual low-frequency hearing who were implanted with a slim straight electrode array.	CI	MP and AP-MP patterns are associated with higher impedance and poorer postoperative hearing. The higher impedances suggest intracochlear fibrosis in these patterns.
Linder et al., [47] 2022	Prospective case series study	Ten patients undergoing CI surgery under local anesthesia without conscious sedation. Intraoperative ECochG monitoring.	CI	Sixty percent of the registered declines in ECochG amplitude were associated with a concomitant attenuation of the subjectively perceived sound. Developments in the ECochG responses matched well with the changes of the sound stimulus perceived by the patients, which supports the applicability of ECochG for preventing insertion trauma.
Dalbert et al., [50] 2021	Prospective cohort	Twelve subjects undergoing CI surgery.	CI	Signals in intracochlear ECochG recordings are larger than signals recorded from an extracochlear location. Residual high-frequency hearing is associated with larger ECochG signal amplitudes.
Valenzuela et al., [48] 2021	Prospective case series	Twenty-two adult CI recipients with various degrees of low-frequency hearing loss.	CI	ECochG responses recorded immediately postoperatively were not associated with 6-month speech perception scores. Mild to moderate relationship between preoperative behavioral audiometric testing and intraoperative ECochG threshold estimates.
Weder et al., [49] 2021	Prospective cohort study	Seventy-three adult patients receiving lateral wall cochlear implant electrode between 2017 and 2023.	CI	Change of ANN/CM index (ratio of auditory nerve neurophonic to cochlear microphonic) linearly correlated with acoustic hearing outcomes 4 weeks after surgery.

### Included studies

Dalbert et al. compared the amplitudes of intra- and extra-cochlear ECochG signals during CI insertion and related these signals to the patients’ preoperative residual hearing [50]. Twelve subjects aged 30–79 with progressive hearing loss were included. The preoperative pure-tone audiogram testing showed a mean hearing threshold at 0.5 kHz of 80 dB HL. ECochG recordings were obtained in a stepwise manner during CI surgery with a custom-made intracochlear electrode from MED-EL and a sterile disposable monopolar needle for the extracochlear electrode. Simultaneous intra- and extracochlear ECochG recordings were obtained in 10 study subjects. Intracochlear ECochG amplitudes were greater in all cases when compared to extracochlear recordings. The researchers hypothesized that larger intracochlear signals could be attributed to their proximity to the auditory hair cells, which are responsible for generating signal responses. However, compared to the intracochlear signals, the extracochlear signals remained more stable in terms of amplitude and phase changes simultaneous to the recorded intracochlear signals. As a result, it was suggested that trauma, injury, or changes in inner ear mechanics were likely not the cause for the spikes in intracochlear signals, but rather the movement of the intracochlear electrode itself. To relate these findings to preoperative hearing ability, residual preoperative hearing and the recorded ECochG amplitudes were correlated but found to be weak to moderate and nonsignificant. The strongest correlation found was between maximum ECochG amplitudes and high-frequency residual hearing. This showed an association between better high-frequency residual hearing and larger amplitudes on ECochG [50]. Due to the short length of the recording electrode not reaching the apex of the cochlea as consistently, it was concluded that the larger amplitudes seen in the high-frequency regions were a result of the electrode length and placement [50]. One limitation of the study was the step-by-step approach (pausing insertion to record ECochG after each electrode entered the cochlea) used in ECochG recordings, which may have resulted in not all signal amplitudes being captured by the electrodes. The results of this study suggest associations between preoperative residual hearing and intracochlear signal amplitude as well as the complexity of interpreting ECochG signals and discerning trauma from movements of the electrode.

Linder et al. compared patients’ subjective hearing to ECochG measurements during CI surgery under local anesthesia without conscious sedation [47]. The goals of the study were to determine if using subjective hearing was a valid method of cochlear monitoring during surgery and to associate changes in sound perception with ECochG signal events. A total of 10 patients who received CIs with low-frequency residual hearing, measured by pure-tone air-conduction thresholds before implantation, were included in the study. Measurements were taken both on the first postoperative day and 2-to-7 months post-implantation. Either a MED-EL 12-channel lateral wall electrode or a 16-channel array by Advanced Bionics were used. In addition to ECochG monitoring during CI surgery, an earphone was used to send a sound stimulus between 250–1000 Hz. The patient was instructed to describe perceived changes in tone. A reported drop in perceived sound stopped the insertion, and the electrode was removed until sound could be heard again. Of the 10 study participants, 5 patients were candidates for electric-acoustic stimulation, which allowed the researchers to determine average postoperative hearing deterioration. Results from the study show that all 10 patients were able to report their subjective sound perception, while only 7 patients had reliable ECochG measurements. In the patients with both ECochG and sound perception metrics, 60% of the 15 recorded drops in ECochG signals were mirrored in subjectively perceived decreases in sound loudness [47]. The researchers also observed that patients who had consistently high ECochG amplitudes during surgery had better postoperative hearing preservation than those with multiple ECochG amplitude drops. Some limitations of this study include the small sample size of 10 patients, the potential for sound pollution from the operating room affecting perceived hearing, and the innate inaccuracy presented in making subjective reports on sound. Overall, assessing intraoperative subjective hearing with this method was valid, and ECochG signals were correlated with sound perception changes. However, ECochG results seem to be more reliable under general compared to local anesthesia.

Bester et al. looked to correlate peri- and postoperative ECochG patterns with four-point impedance measurements and the residual hearing ability of individuals, which could be used as a marker for intracochlear fibrosis and audiometric thresholds after CI surgery [46]. Four-point impedances assess the local impedance within the cochlea by passing a current between two electrodes, while the potential is recorded between two electrodes lying between the stimulating electrodes. Thirty-nine subjects with residual low-frequency hearing who received Nucleus CI422 or 522 implants were included. ECochG recordings were made immediately after CI insertion and 3 months after surgery with hearing preservation assessed before surgery and after 3 months. Four-point impedance measurements were used to determine local resistance within the cochlea adjacent to the recording electrodes. Three different ECochG patterns were detected during the experiment: 56% of subjects had the largest amplitude around the apical peak (AP) or tip of the electrode, 44% had a mid-peak (MP) in the basal region, and at 3 months, 6 individuals or 15% of total participants who started with an AP switched to a MP pattern (AP-MP). MP and AP-MP patterns were correlated with higher four-point impedance measurements, 295 and 347 Ω, respectively, worse postoperative hearing levels, and were therefore associated with increased intracochlear fibrosis when compared to the AP pattern. It was suggested that this intracochlear fibrosis was caused by inflammatory processes within the cochlea due to trauma from contact between the electrode array and the basilar membrane. Measurements of hearing preservation showed stark differences between the three observed patterns. At 3-months postoperatively, mean hearing loss was 12 dB for the AP group, 28 dB for MP, and 35 dB for AP-MP. One important observation from this data was a CM amplitude correlation with preoperative hearing thresholds for the AP and AP-MP groups, but no correlation for the MP pattern. This demonstrates the immediate deterioration of residual hearing in the MP pattern group, likely caused by trauma from the electrode array insertion, and could be utilized as a future predictor for increased postoperative hearing loss after 3 months [46]. However, a limitation of this study is that basilar membrane fixation, stiffening or scarring of the basilar membrane decreasing its ability to move effectively, from CI insertion trauma cannot be directly proven, rather multiple measurements such as ECochG, four-point impedance, and hearing loss thresholds are taken and interpreted together [46]. Future methods to diagnose basilar membrane fixation in patients postoperatively will help to more easily predict long term hearing outcomes. Although it should also be noted that it is unlikely that all of the MP and AP-MP patterns are caused by the same mechanism of trauma. Overall, this study shows how 3-month hearing outcomes can be predicted from the correlation of four-point impedances and ECochG recordings made during implantation, as well as postoperatively.

Bester et al. analyzed 60 adult patients who were operated on by 5 surgeons and received a thin straight electrode by the Cochlear Company (CI422 or CI522). They were randomly assigned to the treatment or control group. The treatment group’s implantation was monitored using ECochG. If there was evidence of a decrease in more than 30% of the CM amplitude compared to the previous maximum, the surgeon would pull back the electrode until the amplitude recovered. If amplitude was not recovered after 3 attempts, the electrode was then advanced. Intraoperative ECochG was recorded with the most apical electrode contact during insertion. Preoperatively, there was no significant difference in hearing thresholds at 0.25 and 1 kHz (low frequency). The intervention group, those that had the electrode readjusted with changes in ECochG amplitude, was found to have significantly better hearing preservation for absolute hearing loss at a pure tone average (PTA) of 0.25, 0.5, and 1 kHz, as well as for relative hearing loss at the same frequency. When looking at each frequency individually, there was only a significant difference at 1kHz. The group that received the intervention had significantly higher rates of CM preservation, 23 in intervention and 11 in the control group. When looking at those with no CM drop and recovered CM drops, the hearing preservation was not significantly different [38]. Both those that had a CM drop in the control and a CM that did not recover had similar means for hearing, but the number of participants was too low for statistical analysis. They did note that larger cochlear size is associated with CM recovery with intervention. This study concluded that withdrawing the electrode after a CM amplitude drop of at least 30% and allowing recovery of the signal leads to improved hearing preservation [38]. This study shows that a drop in ECochG is not associated with irreversible cochlear damage, and that the recoverable drops are likely due to contact with the basilar membrane. The most recoverable drops were seen within the first 20mm of insertion.

Buechner et al. analyzed intraoperative ECochG of 64 adults (>18yo) receiving a Cochlear Limited’s Nucleus CI422 or CI522 with measurable residual hearing at one frequency [28]. Electrocochleography was recorded throughout electrode insertion, and the surgeon adjusted the electrode position (pulled back) with any drops in the CM. No specific amplitude change was used. Of note, full insertion of the electrode was not achieved in patients for whom the CM was recovered with 1–3 electrodes outside the cochlea. The CM results were divided into three groups; growth (no large drops during insertion), fluctuating (some larger drops were seen during insertion, but a response was present at the conclusion of insertion), and total loss (a response was measurable during insertion, but not at the end). Upon analysis of the median hearing loss (HL) for all 47 subjects included in analysis, there were no significant differences [28]. The data was reclassified based on previous findings by Koka et al. and Giardinia et al. to analyze both CM amplitude changes and phase shifts. It was suggested that when a CM amplitude drop happens in conjunction with a phase shift, the drop may not be due to trauma. The reclassification was a type I (no drop >5 dB), type II (> 5dB drop with phase shift), and type III (>5 dB drops without a phase shift). Type III was found to have significantly worse hearing preservation compared to other types (Krustakal-Wallis rank sum with multiple comparison test, p = 0.005 and p = 0.008). There were no significant results between type I and II [28]. The preoperative hearing thresholds were analyzed to determine if the better hearing preservation in type III was due to differences in hearing prior to implantation. No significant differences were seen. It was concluded that those with a constantly increasing CM amplitude, no drops, had the best hearing preservation. The group with fluctuating ECochG results showed no statistically significant correlation with hearing preservation after implantation. Further research is warranted to explore the components of the ECochG signal and their significance during surgery.

Harris et al. studied the impact of an audible intraoperative ECochG alarm on hearing preservation outcomes [37]. The study enrolled 80 adults (>18 years) who had a preoperative PTA of 80 dB or less at 500 Hz. During the procedure, patients were randomly assigned to an audible ECochG alarm “on” and audible ECochG alarm “off”. The 28 surgeons from 10 high volume CI centers were instructed that if the audible “drop” alarm, a knocking sound, was heard, they should consider pausing and possibly pulling back on the electrode until the alarm ceased. The surgeons were instructed to reach full insertion of the electrode for all patients. The mean PTA was 54 dB HL. Of the 28 included patients, 34.5% showed “good” hearing preservation, 22.5% showed “fair” hearing preservation, and 43.5% showed “poor” hearing preservation [37]. They did find a significant difference in the unaided preoperative PTA between the SlimJ and HiFocus Mid-Scala electrodes, though the electrode was chosen independent of study enrollment. The postoperative PTA was not significantly different between the ECochG alarm “on” and ECochG alarm “off” groups. Interestingly, there was also a significant decrease in scalar translocations in those with the “alarm on” group.

Lenarz et al. also analyzed the relationship between intraoperative ECochG and hearing preservation [30]. The study included 68 adult (>18 years) CI recipients who received Mid-Scala or SlimJ electrode having preoperative hearing thresholds of ≤ 90dB HL at 500 Hz. There was no intervention by the surgeon based on ECochG results. The intra operative ECochG results were analyzed with the hearing preservation outcomes to better understand the relationship. The larger ECochG drops were associated with larger PTA changes [30]. However, no correlation between preoperative hearing level and the amount of hearing loss was observed. The study revealed a stronger correlation between ECochG and PTA in patients implanted with the SlimJ electrode compared to those with the Mid-Scala array.

Saoji et al. looked at the relationship between ECochG and postoperative hearing preservation [36]. They analyzed 16 adult (>18 years) patients who had measurable bone conduction (BC) thresholds at 250 and/or 500 Hz. Fifteen had Cochlear Nucleus implants and one had an Advanced Bionics implant. Nine patients had transient drops on the ECochG amplitudes, and seven patients had increasing ECochG amplitude with electrode advancement and a subsequent >30% drop in the amplitude before complete insertion [36]. They also found that patients with preserved BC thresholds immediately postoperatively had decreases 1 month postoperatively. Patients with a large (>30 dB) drop intraoperatively showed threshold shifts immediately postoperatively at 500 Hz BC. Five of the participants had a small ECochG amplitude drop (<30%) and had preserved immediate postoperative hearing thresholds but were found to have a decreased 500Hz BC threshold one month postoperatively. Three patients with small drops did not show this delayed change in hearing. Two had large drops in ECochG amplitude and had no further decrease in hearing thresholds at 500Hz BC one-month post-implantation.

Sijgers et al. used ECochG intraoperatively for real time feedback of cochlear function, specifically looking at the meaning behind amplitude changes, phase shifts, harmonic distortions, and recording location [39]. Their participants included 6 adults (>18 years) undergoing CI surgery at a university hospital, with residual hearing of any level. One participant received a HiFocus V electrode (Advanced Bionics) and the remainder received SlimJ electrodes (Advanced Bionics). Pure tone audiograms were done within 3 months prior to surgery and 4 weeks after. They define 3 hearing preservation patterns: complete preservation (mean low frequency loss ≤ 10 dB), partial hearing preservation (mean low frequency loss > 10dB with some low frequency hearing), and no hearing preservation (complete loss of residual hearing). The study observed a decrease in amplitude, along with a 180-degree phase shift and harmonic distortions, recorded inside the cochlea during the first half of the insertion. However, this decrease in intracochlear amplitude did not correspond to a reduction in the amplitude of the signal measured outside the cochlea [39]. Late intracochlear amplitude decreases did, however, correlate with extracochlear amplitude decreases. They had two participants with complete preservation, two with no preservation, and 4 with partial preservation. The study revealed no correlation between the degree of hearing preservation and changes in ECochG amplitude. Nonetheless, they suggested that the observed drops in amplitude might indicate cochlear trauma.

In O’Leary et al., 109 patients had ECochG recorded after implantation of a slim-straight electrode array (Cochlear Limited Nucleus’s CI 422 or 522 implants) [29]. Each patient had hearing ≤ 80 db at 500 Hz. Of the 95 patients that had interpretable ECochG signals, 66 had an ECochG amplitude drop during implantation. The patients with a drop had poorer hearing pre-operatively when above 1000 Hz. At 3 months, those with an ECochG drop had significantly worse hearing preservation at 250, 500 and 1000 Hz. They determined that this ECochG drop during surgery was predictive of greater relative and absolute loss of residual hearing [29]. This study excluded children, the mean age was > 65 years, but age was not associated with amplitude drops. The article suggests that these findings could ultimately lay the groundwork for utilizing ECochG in surgical decision-making processes. Additionally, the study observed that in half of the patients, hearing worsened between 3 and 12 months, irrespective of any drop in intraoperative ECochG. This demonstrates that ECochG primarily reflects short-term outcomes, and its ability to predict long-term results is limited.

Ulukan et al. studied 62 implanted ears in 46 recipients. The mean age of the recipients was 11 years old; 29 of them had auditory neuropathy spectrum disorder, and 33 had sensorineural hearing loss [41]. In this study, 55 children had CI24RE electrodes, five had CI512 electrodes, and two had CI422 Slim Straight electrodes. They analyzed the differences in postoperative ECochG results between patients diagnosed with Auditory Neuropathy Spectrum Disorder and those with SNHL. There was a difference in the hearing threshold between the two groups, as determined by audiograms at frequencies of 250, 500, 750, and 1500 Hz. CM responses were recorded in 27 out of the 62 ears tested. The findings indicated that the hearing thresholds for the ANSD group were significantly better than those for the SNHL group. Moreover, no correlation was found between ECochG outcomes and behavioral hearing thresholds, contradicting the results of earlier studies [41]. This suggests that CM responses might not effectively predict functional hearing outcomes. However, the study had a small sample size and there was an extended period between the implantation and the recordings.

Tejani et al. conducted a study on 40 subjects who underwent cochlear implantation, dividing them into two groups for comparative analysis, one with stable hearing and the other experiencing hearing loss [42]. The electrode arrays used in this study were all Cochlear arrays (S8/S12/L24 hybrids arrays, CI 422/522/622 slim lateral wall arrays, and CI 624 slim 20 arrays). ECochG postoperatively in each of the groups were measured at 0.5, 1, 3, 6, and 12 months at 250, 500, 750, and 1000 Hz with a special focus on 500 Hz. CM and ANN were determined for each participant at designated time intervals. It was observed that the consistency in the amplitudes of CM and ANN corresponds with the reliability of ECochG assessments across different tests and within the same test, for both groups with normal hearing and those experiencing hearing loss. [42]. There was also a correlation between behavioral and ECochG thresholds, which suggests that ECochG measurements can be a useful tool for monitoring residual hearing.

Walia et al. compared the stability of CM amplitude during insertion with the stability of the post-operative audiogram to determine whether a 250 or 500 Hz stimulus is superior in predicting residual hearing preservation [43]. This study has 48 adult CI candidates that were implanted either with perimodiolar (CI612 or CI632) or slim lateral wall electrode arrays (CI624). The ECochG responses using individual electrodes for 250 and 500 Hz stimuli were measured, as well as shifts in low-frequency pure tone average (LFPTA). A stronger linear correlation between ECochG drops and LFPTA shifts was observed when measured at 250 Hz rather than 500 Hz. Overall, they concluded that a 250 Hz stimulus for feedback is more predictive of hearing preservation than a 500 Hz stimulus [43]. The results of this study are in agreement with the findings of previous studies [29,30]. These three studies collectively demonstrate that patients experiencing a drop in ECochG greater than 30% during insertion tend to have poorer hearing preservation compared to those who do not experience such a drop. However, it is important to note that the latter two studies employed a 500 Hz acoustic stimulus and did not utilize a 250 Hz stimulus.

Valenzuela et al. aimed to determine the relationship between intracochlear ECochG response amplitudes and 6-month speech perception scores, in addition to the relationship between behavioral auditory thresholds and ECochG threshold estimates [48]. They hypothesized that the amplitudes of intracochlear ECochG responses measured immediately after electrode insertion would surpass those recorded at extracochlear sites in historical controls. This could account for the observed greater variability in speech perception scores. Twenty-two adult CI recipients with varying degrees of low-frequency hearing had intracochlear ECochG measurements made immediately after CI electrode insertion and 110 dB SPL tone bursts. Tone bursts were centered at five octave-spaced frequencies between 125 and 2,000 Hz. Results demonstrated no association between intracochlear ECochG response amplitudes and speech perception scores [48]. Nonetheless, the data suggests a mild to moderate relationship between preoperative behavioral audiometric testing and intraoperative ECochG threshold estimates [48]. Performing intracochlear ECochG is highly feasible and results in larger response amplitudes than historical controls at the extracochlear site. The results demonstrate a potential correlation between intraoperative ECochG predicted hearing thresholds and preoperative behavioral hearing thresholds. However, as no association was found between intracochlear ECochG response amplitudes and speech perception scores, it was suggested that performing ECochG before, rather than after, CI insertion may provide a more accurate assessment of a patient’s speech perception potential.

Weder et al. aimed to investigate whether or not particular ECochG components would help in more accurately predicting postoperative hearing outcomes, as steep amplitude drops of the CM signal have been correlated with lower outcomes in residual hearing but maintain a sensitivity and specificity that are too low to use CM signaling as a biomarker [49]. The study included 73 adult patients receiving a lateral wall cochlear implant electrode (CI522 Slim Straight Electrodes). During electrode insertion, rt-ECochG measurements (Real-time ECochG - a method to detect intracochlear potential changes during CI) were performed. Authors calculated a multiple regression analysis for patients with one ECochG event, using the relative acoustic hearing result 4 weeks after surgery as the dependent variable. The independent variables were the CM latency, a ratio of the auditory nerve neurophonic to the CM known as the ANN/CM index, and the CM signal recovery [49]. The change of the ANN/CM index linearly correlated with acoustic hearing outcomes 4 weeks post-operatively. This particular correlation suggested a statistically significant increase in the variance accounted for by the regression model. When monitoring the implantation process with rt-ECochG, the authors concluded that prediction of postoperative hearing thresholds is improved by addition of the ANN/CM index to a model that includes CM amplitude fluctuation.

Walia et al. performed a study with 35 adult patients that have all received their cochlear implants at the same surgical center [44]. The ECochG was measured intraoperatively and at 3 months post-CI. Audiograms were performed at three months postop as well (both pure tone average and low frequency pure tone average). It was observed that ECochG had a moderate-to-strong correlation with LFPTA, and a slightly stronger correlation with PTA [44]. Data was collected utilizing an intracochlear recording device. They also evaluated speech and word recognition capabilities using the consonant-nucleus-consonant (CNC) score. They found that using ECochG for prediction of this test accounted for 59% of the variance in a quiet environment. In this study, background noise was also considered, and it was observed that the predictive value of the ECochG measurement diminished under these conditions.

Suntinger et al. studied 68 subjects, measuring ECochG recordings during and directly after implantation [45]. The participants had various underlying diseases, including idiopathic hearing loss, hereditary, infectious, otosclerosis, exposure to ototoxic substances, and Meniere’s disease. They were implanted with various CIs such as Nucleus CI422, CI522, CI512, CI612, HiRes 90K HiFocus V, HiRes90K, HiFocus Slim-J, and MedEl Flex 28. The hearing outcomes were measured at 250, 500, 750, 1000 Hz. They found that only 48 out of 210 of the recordings had a phase change > 45 degrees [45]. In these ears that also had an amplitude drop of more than 3 dB, they all had a complete or near-complete loss of residual cochlear function. Direct linear correlations between changes in the amplitude of the ECochG signal and the preservation of hearing could not be established. [45]. However, the phase changes had a more significant effect on hearing outcomes than amplitude changes. Subjects exhibiting a phase change greater than 90 degrees experienced more substantial postoperative hearing loss almost without exception, regardless of any amplitude reduction. Thus, a phase change of 90 degrees or more was identified as a reliable indicator of postoperative hearing loss.

Finally, Weder et al. aimed to define he changes that occur in real-time ECochG recordings to make them more useful for predicting post-operative hearing loss and possible surgical interventions to prevent post-operative hearing loss [40]. In this investigation, 55 patients, each with a pre-operative hearing threshold of 100 dB or less at 500 HZ, underwent cochlear implantation with a CI522 slim straight electrode. During implantation, intracochlear ECochG measurements were taken, though no surgical interventions were made to notify the surgeons of any changes. These recordings were analyzed for the occurrence of an ECochG event that would predict post-operative hearing loss after 4 weeks. At least one ECochG event was experienced by 77% of the patients, and each subsequent event increased the risk of greater post-operative hearing loss, while patients with no ECochG events had better preserved residual hearing [40]. Patients with only one ECochG event showed many different levels of postoperative hearing. Additionally, it was observed that bigger amplitude drops on one-drop patients are correlated with worse hearing preservation, although it did not reach statistical significance [40]. The study also found trends related to the timing of ECochG events, where those that occur earlier in the surgery demonstrated a relative hearing loss greater than 50% at 500 Hz. Also, drops that occur when the electrode is moved from its mid-point to the first white marker or as the fascia is placed on the round window, both were associated with improved preservation of residual hearing. They also explored how signal recovery after one ECochG event affects hearing preservation but found no significant correlation.

## Discussion

Electrocochleography was initially developed in 1930 following the recording of CM potentials in animals [51]. Shortly after, ECochG began utilizing electrodes that were inserted into the middle ear through the tympanic membrane for recordings at the round window or placed via the stapes during stapes surgery referred to as extracochlear ECochG. Although extracochlear ECochG is less invasive, it has challenges in obtaining reliable and reproducible recordings. Research indicates that waveforms were successfully recorded in 52–100% of patients, highlighting the variability in its effectiveness [52–55]. Intracochlear ECochG has a much more recent inception, with the first documented use in 2014 by Calloway, who utilized a lateral wall CI array for recording [56]. More recently, the same electrode array used for CIs can also be used to record intracochlear ECochG, making the workflow simpler [57]. When recording from within the cochlea, the amplitude of recorded potentials is larger, allowing for easier analysis of changes [54,58]. Currently, the most common uses of ECochG are for Meniere’s disease, where an elevated SP/AP ratio is thought to indicate endolymphatic hydrops, and to determine sensory structure versus neural origin of hearing loss [59,60]. This review suggests that ECochG has some utility to predict preservation of residual hearing post-cochlear implantation. Electrocochleography (ECochG) is a technique that measures the electrical potentials generated by the cochlea and auditory nerve in response to sound stimuli. In recent years, intraoperative ECochG has emerged as a promising tool for real-time monitoring of cochlear function during cochlear implant (CI) surgery. This technique provides immediate feedback on the cochlea’s response to the electrode, offering surgeons valuable insights during the insertion process. ECochG can help detect potential trauma to cochlear structures, guide electrode placement to minimize damage, and predict postoperative hearing preservation outcomes. Understanding the relationship between intraoperative ECochG measurements and postoperative hearing outcomes is crucial for developing strategies to maximize hearing preservation in CI recipients. This knowledge has the potential to lead to improved surgical techniques, better electrode designs, and ultimately, better hearing outcomes for CI users. As research in this field continues to advance, ECochG is becoming an increasingly important tool in the pursuit of optimal cochlear implant outcomes.

While ECochG yields four basic potentials for analysis of the inner ear (CM, CAP, SP, ANN), the studies included in this review primarily utilized the CM potential or difference curves, which are thought to be synonymous [39]. This aligns with expectations, considering that most of this signal is derived from the outer hair cells of the OC, and the signal amplitude is directly proportional to the displacement of the OC, encompassing both inner and outer hair cells [61,62]. The amplitude of the ECochG recorded potentials was a key focus in all of the included studies. Generally, a strong and stable CM amplitude, particularly without any significant drops, was associated with improved postoperative residual hearing, as evidenced in four studies [28,46,47,50]. Prior studies have noted the utility of the CM potential in determining the presence of cochlear damage [63,64]. In addition to CM, CAP has been studied in preliminary animal studies as being useful in detecting cochlear damage [64]. CAP is an entirely neural response, which represents all of the action potentials of the auditory nerve summed [65]. CAP tends to be most reliable at high frequencies, which is where hearing loss commonly occurs. Due to the unreliability of CAP at low frequencies, it is not the preferred method for monitoring hearing function during cochlear implant surgery. While CM was preferred in these studies, further investigation into the utility of other recorded potentials could prove useful in creating objective measures for ECochG as a measure of residual hearing and cochlear damage intraoperatively.

The presence of CM amplitude drops was seen to be correlated with worse hearing preservation postoperatively in five studies. Saoji et al., O’Leary et al., and Walia et al. defined a significant drop as a 30% reduction in ECochG amplitude, whereas the studies by Lenarz et al. and others did not set a specific threshold for amplitude changes [29,30,36,43,44]. In terms of measuring hearing preservation, Lenarz et al. and Walia et al. employed pure tone audiometry (PTA), Saoji et al. used bone conduction measures. Interestingly, Saoji et al. found that even when hearing was preserved immediately after surgery, hearing thresholds tended to deteriorate one month postoperatively, irrespective of the initial postoperative hearing status. This is a dilemma that neurotologists have faced since the advent of cochlear implants. Cochlear implants initially create damage to the auditory structures when drilling near the RW, creating the cochleostomy, or when electrode insertion damages the delicate cochlear structures [66]. All of these initiate immediate inflammatory, necrotic, and apoptotic responses which contribute to the loss of residual hearing [67,68]. Next fibrosis ensues in the cochlea due to chronic inflammation, leading to delayed residual hearing loss [69]. Mitigating the initial trauma, via intra operative ECochG, could mitigate the initial inflammatory reaction and possibly decrease the overall inflammatory response and subsequent residual hearing loss.

Currently, there is no universally agreed-upon standard for what constitutes a clinically significant change in ECochG amplitude, though several studies have adopted the 30% reduction benchmark. Further investigations are warranted to determine the critical percentage change in amplitude that would be both sensitive and specific enough, confirming if a 30% threshold change is appropriate. Several studies used ECochG intraoperatively and intervened based on the data collected. Bester et al. retracted the electrode and replaced it after experiencing a 30% drop in the (EChochG) amplitude. It was observed that subjects receiving this intervention had better hearing preservation for absolute and relative hearing loss at 0.25, 0.5 and 1kHz [38]. Hearing preservation did not, however, differ between those with a recovered and no drop. Contrary to this, other studies all noted no difference in hearing outcomes for those that received an intraoperative intervention with those who did not [37,39,41,45]. Within the studies that intervened during insertion based on ECochG amplitudes, there were differences in insertion depth. Buechner et al. did not always achieve full insertion of the electrodes, while Harris et al. always achieved full insertion. Electrode insertion techniques are used to decrease intracochlear trauma and improve outcomes, referred to as soft surgical techniques. These include leaving the cochlea open for the shortest amount of time and slow insertion of the electrode with immediate cessation of advancement if any resistance is felt. The depth of insertion is a subject of significant discussion. It is advised to aim for full insertion during the procedure to attain optimal speech and hearing outcomes [70,71]. This is in part due to the assumption that the disease process that caused hearing loss will continue and eventually impact the low frequency hearing located in the apex. However, the evidence regarding the impact of insertion depth on hearing outcomes is mixed. This ambiguity is attributed to the limited size of study samples and differences in electrode types [72]. There is some indication that full insertion may increase the chances of scalar translocation [73]. While full insertion might enhance speech perception, it could also potentially worsen hearing preservation [74,75]. Strictly adhering to ECochG feedback in an effort to reduce trauma may necessitate stopping electrode insertion prior to full insertion, potentially compromising the optimal electrode placement or limiting ECochG applicability if full insertion is completed. These findings are not definitive, and there’s a need for larger-scale studies to clarify these potential correlations [73,76].

ECochG in the reviewed studies was recorded via two different techniques: intracochlear and extracochlear signal monitoring. Intracochlear signals were primarily recorded from the inserted CI electrode, while extracochlear signals were obtained from outside the cochlea. Across the studies, intracochlear ECochG recordings had larger amplitudes than extracochlear recordings, whereas extracochlear recordings were overall more stable over the course of a recording [50]. Intracochlear ECochG was also able to detect more amplitude changes and fine structure components than the extracochlear ECochG, particularly during the initial phase of electrode insertion [39]. Researchers proposed multiple potential causes for the higher amplitude and number of intracochlear recordings. Higher amplitudes were attributed to the intracochlear electrode being physically closer to the area of recording, the OC hair cells, than the extracochlear electrode [48]. The greater number of amplitude changes with intracochlear recordings was thought to be a result of the movement of the intracochlear electrode inside the cochlea compared to the stationary extracochlear electrode sitting outside the cochlea. Additionally, middle ear effusion during surgery could cause non-trauma related amplitude changes in the intracochlear recordings by moving the electrode [50]. While some amplitude spikes could be outliers or attributed to movements of the electrode, the presence of higher intracochlear amplitudes could allow for the detection of smaller traumas that, otherwise, could have been missed by solely using an extracochlear electrode [43]. By incorporating both intracochlear and extracochlear electrodes, the cochlea can be monitored from dual viewpoints, offering surgeons comprehensive ECochG data before, during, and after the cochlear implantation, while requiring only minimal extra steps in the CI surgery process. The precise system used for ECochG measurement is largely dependent on both the implant selected and the research team leading the study.

There is a consistent trend that suggests the potential benefits of using ECochG during cochlear implantation. However, the current evidence is not strong enough to establish this with certainty and have electrocochleography be proposed at the standard of care during implantation. Therefore, more comprehensive research is essential to solidify these preliminary findings. Future studies should aim to provide more definitive data, possibly through more controlled settings, and longer follow-up periods. This additional research is crucial not only to confirm the initial observations but also to understand the full scope and limits of ECochG’s effectiveness in CI surgeries. A better knowledge regarding the utility of ECochG during CI surgery will facilitate in making more informed decisions about the integration of ECochG into standard clinical practices for cochlear implantation, potentially leading to enhanced patient outcomes.

### Limitations

Many of the included studies in this systematic review had limitations. The studies involving the use of ECochG during cochlear implantation generally have small sample sizes. This limited participant number poses significant challenges in drawing definitive conclusions, as the smaller data set may not adequately represent the broader population. Consequently, the findings from these studies should be interpreted with caution as the results might not be sufficiently robust or generalizable to form conclusive evidence applicable to all cases of cochlear implantation, particularly children.

The variability in the types of electrodes used during cochlear implantation with ECochG adds a layer of complexity to the analysis of data. Different electrode types can produce varying results due to their specific design and interaction with the cochlea. The choice of electrode is a critical decision made by clinicians based on several factors. These factors include the patient’s unique anatomical structure, the underlying etiology of their hearing loss, and the capabilities of the available devices selected by the patient and clinical team in the hospital. This individualized approach to electrode selection, while beneficial for tailored patient care, introduces a range of variables into the data. As a result, comparing outcomes across different studies becomes challenging, as each study may use a different combination of electrode types, influenced by patient-specific considerations and clinician preference. This diversity in electrode selection underscores the need for careful interpretation of study results and suggests a potential area for standardization or further research to better understand the implications of electrode type on ECochG outcomes during cochlear implantation. Future studies that incorporate almost equal numbers of all electrode types are warranted, which could significantly enhance our understanding of the role of intraoperative ECochG in enhancing hearing preservation during cochlear implantation. Such comprehensive research would provide a more balanced and representative analysis of the different electrode types as well as their impact on auditory outcomes. By including a diverse range of electrodes in these studies, researchers could gain deeper insights into how each type interacts with the cochlea and influences the effectiveness of ECochG. This approach would allow for a more nuanced comparison of results, helping to identify which electrode types are most conducive to preserving residual hearing when used in conjunction with intraoperative ECochG. The insights gained from these large-scale studies could be instrumental in refining surgical techniques, selecting appropriate electrodes, and tailoring individual treatment plans to maximize hearing preservation for implanted individuals.

## Conclusions and future directions

The use of intraoperative ECochG monitoring during CI surgery is increasingly recognized as a tool to enhance surgical outcomes, particularly regarding the preservation of patients’ residual hearing [25–30]. Implanted individuals with preserved residual hearing often experience better speech comprehension and a richer auditory experience, especially in noisy environments [19–21]. One of the primary advantages of intraoperative ECochG monitoring is the enhancement of surgical precision. This technology provides real-time feedback on the status of the cochlea during the implantation process. Such immediate insights allow surgeons to insert the electrodes while minimizing potential trauma to the cochlea. This precise placement is crucial for preserving as much residual hearing as possible, a key concern for patients undergoing CI surgery who still retain some natural hearing ability. Through this review, we see that changes in ECochG amplitude, particularly when not associated with a phase shift, may correlate with decreased residual hearing.

Additionally, ECochG monitoring provides immediate assessment and confirmation of the functionality of the CI device. This immediate validation is crucial for ensuring that the device is working as intended and stimulating the cochlea effectively. It reduces the likelihood of postoperative complications related to device malfunction, thereby reducing the need for revision surgeries. The insights gained from intraoperative ECochG are not only beneficial during the surgery but also extend to postoperative care. The data obtained can guide audiologists in the rehabilitation process, particularly in the programming of the CI, which can impact its outcomes [77–79]. Customizing the CI settings based on individual cochlear response patterns observed during surgery can lead to more effective and personalized auditory rehabilitation for the patient.

Despite advancements in the field of ECochG to be used as a diagnostic tool for predicting preservation of residual hearing post-CI, there are still significant research gaps. These include the need for standardized ECochG protocols and recording systems and a consensus on what constitutes clinically significant changes in ECochG readings, to gain a deeper understanding of long-term postoperative outcomes. The diversity of patient populations and implant types in the studies underscores the necessity for broader research to encompass a wide range of demographic and clinical scenarios. Additionally, the integration of ECochG with other monitoring techniques, the exploration of the impact of various surgical methods, and the economic and accessibility aspects of using ECochG in different healthcare settings are areas that needs to be explored in further investigation. Exploring the use of ECochG in different patient populations, especially those with atypical cochlear anatomy or different etiologies of hearing loss, could be beneficial. Currently, most of the research focuses on standard patient profiles, leaving a gap in understanding how ECochG can be optimized for diverse patient groups. There is a pressing need to focus on patient-centered outcomes and to unravel the underlying biological mechanisms of ECochG changes observed during CI surgeries. Addressing these research gaps could significantly advance the field, leading to improved surgical practices, better patient outcomes, and a deeper understanding of the role of intraoperative ECochG in CI surgeries.

## Supporting information

S1 Checklist PRISMA 2020 checklist. (PDF)

S1 Data A comprehensive spreadsheet documenting each article collected for this manuscript. (XLSX)
